# Genetic Diversity of Hepatitis A Virus in China: VP3-VP1-2A Genes and Evidence of Quasispecies Distribution in the Isolates

**DOI:** 10.1371/journal.pone.0074752

**Published:** 2013-09-17

**Authors:** Hao Wang, Huihui Zheng, Jingyuan Cao, Wenting Zhou, Yao Yi, Zhiyuan Jia, Shengli Bi

**Affiliations:** National Institute for Viral Disease Control and Prevention, Chinese Center for Disease Control and Prevention, Beijing, China; Nanyang Technological University, Singapore

## Abstract

Hepatitis A virus (HAV) is the most common cause of infectious hepatitis throughout the world, spread largely by the fecal-oral route. To characterize the genetic diversity of the virus circulating in China where HAV in endemic, we selected the outbreak cases with identical sequences in VP1-2A junction region and compiled a panel of 42 isolates. The VP3-VP1-2A regions of the HAV capsid-coding genes were further sequenced and analyzed. The quasispecies distribution was evaluated by cloning the VP3 and VP1-2A genes in three clinical samples. Phylogenetic analysis demonstrated that the same genotyping results could be obtained whether using the complete VP3, VP1, or partial VP1-2A genes for analysis in this study, although some differences did exist. Most isolates clustered in sub-genotype IA, and fewer in sub-genotype IB. No amino acid mutations were found at the published neutralizing epitope sites, however, several unique amino acid substitutions in the VP3 or VP1 region were identified, with two amino acid variants closely located to the immunodominant site. Quasispecies analysis showed the mutation frequencies were in the range of 7.22x10^-4^ -2.33x10^-3^ substitutions per nucleotide for VP3, VP1, or VP1-2A. When compared with the consensus sequences, mutated nucleotide sites represented the minority of all the analyzed sequences sites. HAV replicated as a complex distribution of closely genetically related variants referred to as quasispecies, and were under negative selection. The results indicate that diverse HAV strains and quasispecies inside the viral populations are presented in China, with unique amino acid substitutions detected close to the immunodominant site, and that the possibility of antigenic escaping mutants cannot be ruled out and needs to be further analyzed.

## Introduction

Hepatitis A virus (HAV) infection is the most common cause of acute viral hepatitis throughout the world, and remains a significant health problem worldwide. It is spread largely by the fecal-oral route, and contaminated water and food frequently cause community-wide outbreaks [1,2,3,4]. HAV infection has also been spread by contaminated factor VIII and sharing of contaminated needles [5,6]. HAV can lead to a variety of clinical presentations, ranging from asymptomatic infection to fulminant fatal disease [7].

HAV is the only member of the genus Hepatovirus within the family Picornaviridae. The viral genome consists of a 7.5kb, positive-strand RNA with a single long open reading frame (ORF). The ORF of 2,227 amino acids is organized into three function regions termed P1, P2 and P3. P1 encodes the capsid polypeptides VP1-VP4, and P2 and P3 encode non-structural polypeptides. The ORF is preceded by a 5’ untranslated region (UTR) and is followed by a 3’ UTR with a short polyA tail. Only one serotype of HAV has been identified worldwide so far [7]. In natural infection HAV appears to elicit antibodies directed predominantly towards one conserved immunodominant neutralization region. This collection of overlapping epitopes appears to be conformation dependent and distributed over the VP1 and VP3 proteins. Studies of murine monoclonal antibody binding have identified multiple epitopes within this region, and there is evidence for a second, possibly independent site [8,9,10].

Despite this antigenic uniformity, HAV displays a modest degree of genetic diversity. Six genotypes of HAV have been described based on the nucleotide sequence of the VP1-2A or VP1 region [11,12,13]. Genotypes I, II, and III are found in humans, and each of them is further divided into subgenotypes A and B. Recently subgenotype IC isolated from Peru and Spain has been proposed [14]. Genotype I is most prevalent worldwide, and sub-genotype IA is more common than IB. Most of the remaining human HAV strains segregate into genotype III. Genotype IV-VI are detected in simians [13].

The mutation rate of HAV seems to be relatively low, probably as a result of the strict structural constrains of the viral capsid and a restricted codon usage [15,16]. In spite of this relatively low mutation rate the distribution of quasispecies within clinical isolates has been documented [17,18]. It appears that HAV replicates as complex dynamic variety of mutants or quasispecies within the HAV strains. The high degree of conservation of the capsid amino acid sequence could result from negative selection of mutants and convergence of consensus or average sequences [15,17]. HAV co-infection and vaccine escape variants have also been documented, providing the opportunities for recombination and the emergence of new antigenic variants of HAV [19,20].

China is a developing country and exhibits an intermediate to high prevalence of HAV infection, the reported incidence of hepatitis A in China from 2004 to 2007 was 7.2/100 000, 5.6/100 000, 5.4/100 000, and 5.9/100 000, respectively. In these period, between 68 667 and 93 587 cases of hepatitis A have been reported, and the ratios of cases in outbreaks were 2.4%, 2.8%, 2.7% and 3.9% among the reported hepatitis A cases. Children under the age of 10 continue to have a high disease incidence [21,22,23]. Whilst a variety of HAV isolates have been detected in the country, little information is available about the genetic diversity of circulating HAV. Molecular epidemiological analysis is important for understanding the origin of HAV outbreaks, the patterns of HAV transmission and for the better control of the disease.

The aim of this study was to characterize the genetic diversity of the HAV strains circulating in China. The outbreak cases with identical sequences in VP1-2A junction region were selected, the sequences of the core protein VP3-VP1-2A genes were further analyzed, and then the quasispecies distribution in the clinical samples were measured, which could be helpful to provide important biological information about the hepatitis A virus.

## Materials and Methods

### Ethics Statement

HAV is a notifiable disease in China, and the pathogenic surveillance of HAV without disclosing personal information is required by the Law of the People’s Republic of China on the Prevention and Treatment of Infectious Diseases. The data and samples used in this study were obtained as part of this program and according to this law. No identifying patient data was used in this study. The requirement for written informed consent was waived. This study was approved by the second session of the Ethics Review Committee at the Chinese Centre for Disease Control and Prevention.

### Samples

We have analyzed the short VP1-2A junction region of the HAV strains circulating in China from 2003-2008 [23]. This study selected a small part of the hepatitis A outbreak cases that shared identical sequences in the VP1-2A junction region reported from 2003 to 2010 in China, and further analyzed long genomic regions covering the complete VP3-VP1-2A genes.

A total of 42 serum samples which tested positive for anti-HAV IgM were selected and analyzed. They were collected from outbreak cases as acute viral hepatitis A from patients aged from 2 to 32 years. The cases originated from Hebei, Henan, Ningxia and Xinjiang provinces; and samples in some provinces were collected from more than one city or county (Table 1). One serum sample was taken per case. To identify anti-HAV IgM antibodies, an Anti-HAV IgM Kit (Wantai Diagnostics, Beijing, China) was used. Serum samples were aliquoted and stored at -20°C until RNA extraction.

**Table 1:** Abbreviate list of HAV isolates from China used in this study.

Isolates code	Number of isolates	Location(city, province)	Year	Genotype
SjzHbcode.05	5	Shijiazhuang, Hebei	2005	IA
SjzHbcode.07	1	Shijiazhuang, Hebei	2007	IA
PdsHncode.09	2	Pingdingshan, Henan	2009	IA
PyHncode.03	1	Puyang, Henan	2003	IB
QyHncode.10	2	Qinyang,Henan	2010	IA
XxHncode.08	3	Xinxiang, Henan	2008	IA
XxHncode.08	1	Xinxiang,Henan	2008	IB
XxxHncode.09	1	Xinxiangxian,Henan	2009	IA
PyNxcode.07	12	Pengyang, Ninxia	2007	IA
TxNxcode.07	3	Tongxin, Ninxia	2007	IA
XjNxcode.07	4	Xiji, Ninxia	2007	IA
HtXjcode.06	5	Hetian, Xinjiang	2006	IA
LpXjcode.06	2	Luopu, Xinjiang	2006	IA

### RNA Extraction and RT-PCR

Viral RNA was extracted from 140µl serum samples with microspin columns (QIAamp Viral RNA mini kit; Qiagen, Valencia, Calif., USA). The cDNA was prepared by adding 10µl of the extracted RNA to 25µl RT mix with AMV reverse transcriptase according to the manufacturer’s instructions (Promega, Madison, WI, USA). The complete VP3, VP1 and VP1-2A junction region of the HAV genomes were amplified by nested PCR with exTaq polymerase (Takara, Dalian, China) according to the manufacturer’s instructions.

The primers were designed and selected according as previously described [11,12,24], the positions were numbered according to the complete nucleotide sequence of HAV HM175 [Genbank number M14707]. PCR was carried out as described: incubation at 94°C for 5 min, amplification for 35 cycles, with 1 cycle consisting of denaturing for 30 sec at 94°C, annealing for 30 sec at 50°C, and elongation for 60 sec at 72°C, followed by a final extension at 72°C for 7 min [15,23]. The primers used for reverse transcription, nested PCR (RT-PCR) and sequencing are listed in Table 2.

**Table 2:** Oligonucleotide primers used in RT-PCR and sequencing.

Primer	Nucleotide position(nt)^a^	Primer sequence(5’–3’)	Orientation	Reference
VP3F1	1419-1439	GCTAGGTTTACAGATTTGGAG	Forward	This study
VP3R1	2408-2388	TGTCTCAGGCACTTTCTTTGC	Reverse	This study
VP3NF2	1452-1472	ACTCCTCTTTCTACACAGATG	Forward	This study
VP3NR2	2399-2379	CACTTTCTTTGCTAAAACTGG	Reverse	This study
VP1F1	2167-2192	GTTTTGCTCCTCTTTATCATGCTATG	Forward	[12]
VP1R1	3384-3363	CATCCATCTCAAGAGTCCACAC	Reverse	This study
VP1NF2	2181-2200	TATCATGCTATGGATGTTAC	Forward	This study
VP1NR2	3286-3267	TTCATTATTTCATGCTCCTC	Reverse	[12]

^a^ Position related to the genome of HAV strain HM175 (M14707)

PCR products were visualized by UV light in 1.0% agarose gel stained with Gel Red, purified, sequenced in both directions using the ABI Prism Big Dye Terminator Cycle Sequencing Ready Reaction Kit (Applied Biosystems) and an automated sequencer (ABI model 373 or 377; Applied Biosystems, Foster City, CA, USA). Sequences obtained were then edited and analyzed.

### Sequences Analysis

The entire VP3, VP1 and VP1-2A junction region nucleotide sequences of the HAV genomes were compared with HAV sequences deposited in GenBank using the BLAST program, edited with BioEdit and aligned with reference strains from GenBank representing different genotypes and geographical areas using MEGA version 5. Neighbor-joining (NJ) trees were constructed using the Kimura two-parameter method. The reliability was assessed by bootstrap resampling (1,000 pseudoreplicas), only bootstrap values above 80% are shown on the phylogenetic trees. These methods were implemented with program from the MEGA 5 software [25].

The predicted amino acid sequences of the VP3-VP1-2A regions of the HAV genome were also compared with both one another, and with previously published representative reference strains in Genbank, using the MEGA 5 software [25].

### Natural selection analyses

The nonsynonymous and synonymous substitution rates (dN and dS, respectively) were computed to estimate the natural selection pressure on each VP3-VP1-2A codon of HAV, to identify the amino acid sites as evidence of negative, neutral or positive selection, and the overall average dN/dS ratio was calculated using the p-distance method as described by Nei and Gojobori [26] and implemented in the MEGA 5 package [25]. If dN/dS >1 a codon was positively selected, and if <1 then negatively selected. If dN/dS =1, a codon was called neutrally selected [26,27].

### Clonal Variability of the isolates

3 HAV isolates derived from the serum samples of different patients were analyzed. In each sample a mean of 20 molecular clones were analyzed. From a single reverse transcription (RT)-PCR amplification of a fragment of the complete VP3 (738bp) or VP1-2A (1080bp) regions which span sequences encoding the main antigenic sites of HAV [9,10]. Purified VP3 or VP1-2A fragments were cloned into T-vector PMD19 (Takara, Dalian, China), DNA ligations were performed overnight at 16°C using T4 DNA ligase and transformed in *Escherichia coli* DH5α, and transformant clones were screened by the standard white/blue β-galactosidase colorimetric reaction, plasmid DNA from each selected clone was purified by using the Wizard Plus SV Minipreps Kit (Promega, Madison, WI, USA) and confirmed by PCR. Multiples clones of HAV amplicons from both strands of DNA were sequenced by use of an automated sequencer (ABI model 373 or 377; Applied Biosystems, Foster City, CA, USA).

The quasispecies complexity was analyzed by calculating the mutation frequencies of both nucleotide and amino acid sequences and the Shannon entropy in the analyzed isolate clones. The nucleotide mutation frequency was calculated as the total number of mutations divided by the total number of nucleotides sequenced, while the amino acid mutation frequency was calculated as the total number of nonsynonymous mutations divided by the number of amino acids encoded in the sequence analyzed. Normalized Shannon entropies were calculated according to the following formula S_N_ = -[∑_i_ (p _i_ lnp_i_)]/lnN, in which p_i_ is the proportion of each sequence in the mutant spectrum and N is the total number of sequences compared; the normalized Shannon amino acid entropy was calculated as S_NA_= -[∑i (qi lnqi)] /lnN, where q_i_ is the frequency of each amino acid sequence of the mutant spectrum and N is the total number of sequences compared. S_N_ or S_NA_ range from 0 to 1, representing no diversity and maximum diversity respectively [15,17].

### Nucleotide Sequence Accession Numbers

From GenBank, 15 published HAV strains were chosen for comparative phylogenetic studies, the reference sequences Genbank accession numbers are:

GBM(X75215), LU38(AF357222), DL3(AF512536), FH3(AB020569), LY6(AF485328), H2(EF406357), AH1(AB020564), AH2(AB020565), FH1(AB020567), HM175(M14707), BCN70(HQ401240), Shellfish08-106(HQ401253), CF53(AY644676), SLF88(AY644670); AGM27 (D00924).

8 nucleotide sequences of the HAV isolates from China (selected with heterogeneity) in this study have been deposited in GenBank under the following accession numbers: KF006840-KF006847.

## Results

### Genotyping with the VP1-2A junction region, complete VP1 and VP3-VP1-2A regions

**Figure 1:**
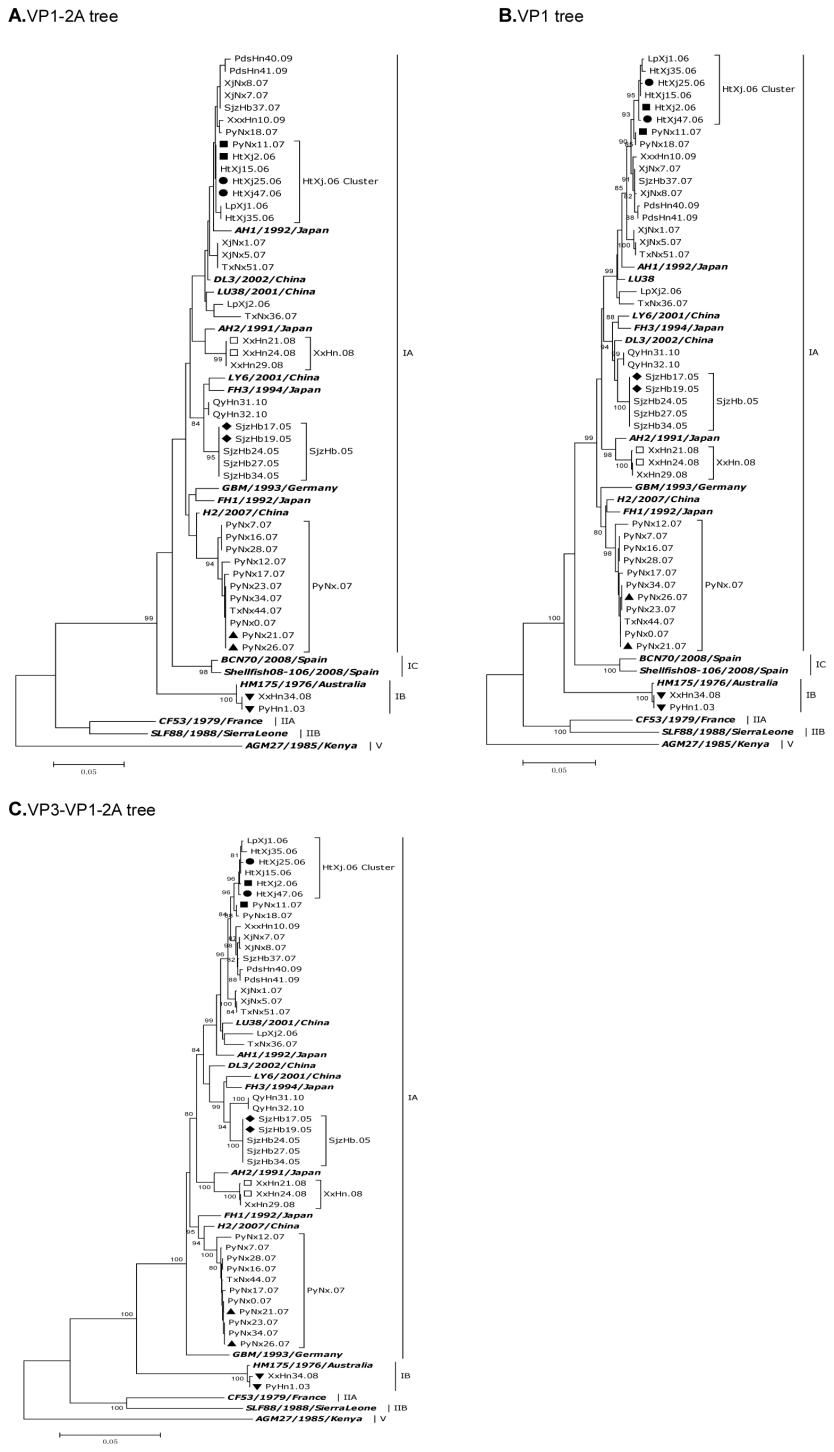
Phylogenetic analysis of HAV sequences isolated in China using Kimura’s two-parameter model. The VP1/2A junction region (A), complete VP1 (B) and VP3-VP1-2A (C) sequences were used for genotyping (see Table 1 for geographical location and year of isolation. In figure 1 (C) the IC subgenotype was not included for the lack of long enough available sequences). **I** indicated genotypes or subgenotypes; bold italic showed reference strains reported previously from Genbank. Numbers beside the branches indicate bootstrap percentages obtained after 1000 replications of bootstrap sampling. Bars show distances. ●▲◆■▼□ represent isolates with identical sequences at the VP1-2A junction region respectively, which showed heterogeneity at the complete VP1 or VP3-VP1-2A regions.

The complete VP3-VP1 plus partial 2A regions of HAV RNA were amplified from the clinical serum samples from among the selected hepatitis A outbreak cases. A total of 42 VP3 sequences (each 738bp in length) and 42 VP1-2A sequences (each 1080bp in length) were obtained from these 42 amplicons. Phylogenetic analysis of the most common VP1-2A junction region (320bp in length) indicated circulation of both sub-genotype IA and IB in China, as shown in Figure 1. A, with of 40 isolates grouped into sub-genotype IA diverging by roughly 0-6.0% when compared pairwise with the IA Chinese HAV isolates and with the reference IA strains from Genbank, 2 isolates grouped into sub-genotype IB diverging by roughly 0-0.4% when compared pairwise with the reference IB strains and with the IB Chinese HAV isolates. We can conclude that a diversity of HAV strains are circulating in China, as has been previously reported [23].

The recently proposed subgenotype IC [14] isolated from Peru and Spain was also included in the phylogenetic analysis. Results indicated that the isolates from China in this study did not belong to the new IC subgenotype based on the current genotyping methods.

When the partial VP1-2A junction region was genotyped several identical or closely related HAV sequences were detected in isolates of temporally and geographically similar origin, e.g. SjzHb17.05 and SjzHb19.05. However, identical or closely related sequences were also detected in some samples of geographically distant origin, e.g. PyNx11.07 and HtXj2.06. In order to further confirm the genetic identity of HAV isolates derived from specific outbreaks, the sequence of the entire VP1 (900bp in length) and the VP3-VP1-2A (1731bp in length) regions were further analyzed in all 42 samples. As shown in Figure 1. B and C, the same genotyping results as the partial VP1-2A junction region were obtained in this study, although some differences did exist.

Some isolates derived from the same outbreak share an identical VP1-2A junction region sequence (e.g. SjzHb17.05 and SjzHb19.05 Figure 1. A), the genetic similarity was confirmed by large fragment analysis (Figure 1. B and C). In contrast the closely related sequences derived from some isolates collected in the same area and year, e.g. PyNx21.07 and PyNx26.07 with identical small VP1-2A fragment exhibited some minor variation in the entire VP1 or VP3-VP1-2A genes (Figure 1). Also some of the isolates collected at different sites in different years share identical VP1-2A junction region sequences (e.g. PyNx11.07 and HtXj2.06) exhibited some variations in the entire VP1 or VP3-VP1-2A genes, but were slightly more variable when compared with the isolates collected from the same outbreak (Figure 1).

### Amino Acid Analysis of HAV Isolates

The predicted amino acid sequences of the VP3-VP1-2A regions (577 amino acids) of the 42 HAV isolates were compared with each other and with previously published strains deposited in GenBank representing sub-genotype IA (DL3, LU38, LY6, H2, AH1, AH2, FH1, FH3, GBM), IC (BCN70, Shellfish08-106), IB (HM175), sub-genotype IIA, IIB, and genotype V [9,10,20]. Although some nucleotide sequences varied in the VP3-VP1-2A regions in these isolates, the amino acid substitutions in these regions were very limited. No amino acid mutations were found at the published neutralizing epitope sites, while several amino acid differences were detected, with two unique amino acid variants (LpXj1.06, VP1-115 and HtXj25.06, VP1-164) closely located to the immunodominant site (Table 3). Moreover, those patients were around 2-9 years old, and had not been vaccinated.

**Table 3:** The amino acid substitutions in the consensus sequences of VP3 and VP1 regions observed in this study and part of the published neutralizing sites from references.

		Changed/Consensus	Published	
Isolates	Position^a^	amino acids	neutralizing sites	Position^a^
HtXj25.06	VP3-124	Asn/Thr	Pro [7,15]	VP3-65
	VP1-164	Pro/Leu	Asp[7,15]	VP3-70
HtXj35.06	VP3-125	Arg/Lys	Ser [7,15]	VP3-71
	VP1-211	Met/Val	Gln [7,15]	VP3-74
LpXj1.06	VP1-115	Leu/Ser	Ser [7,15]	VP1-102
LpXj2.06	VP1-228	Met/Leu	Asn[7,15]	VP1-104
PdsHn40.09	VP3-196	His/Tyr	Lys [7,15]	VP1-105
	VP1-272	Ile/Thr	Ser [7,15]	VP1-114
XxHn34.08	VP1-253	Gly/Glu	Val [20]	VP1-166
			Trp [20]	VP1-170
			Val [7,15]	VP1-171
			Ala [7,15]	VP1-176
			Lys [7,15]	VP1-221
			Gln [7,15]	VP1-232

^a^ Position related to the genome of HAV strain HM175 (M14707).

### Natural selection analysis

In this context, if the protein-coding region is undergoing positive selection, the rate of non-synonymous substitution (dN) observed in that region will be greater than the rate of synonymous substitutions (dS) (dN/dS>1). Conversely, if dN is found to be less than dS (dN/dS<1), then negative selection is operating in that particular coding region. If the gene experience genetic drift, accumulating mutations that neither improve nor reduce the viral fitness (i.e. they are neutral), the dN will be equal to dS (dN/dS=1) [27].

The frequencies of synonymous (dS) and non-synonymous (dN) were calculated using the Nei-Gojobori method with MEGA 5.0 package based on the HAV VP3-VP1-2A gene regions (577codons), and none codon was found under positive selection pressure. The calculated mean nonsynonymous/synonymous distance ratio was 0.014, which is less than 1, indicating there is a clear negative selection of those replacements for HAV core proteins.

### The mutant quasispecies spectrum in clinical isolates of HAV

In order to explore the distribution of quasispecies within these isolates, the entire VP3 and VP1-2A regions were cloned from 3 clinical samples: SjzHb19.05, HtXj15.06 and PyNx21.07. A total of 120 clones, including 60 VP3 and 60 VP1-2A clones, were obtained from these amplicons.

**Figure 2:**
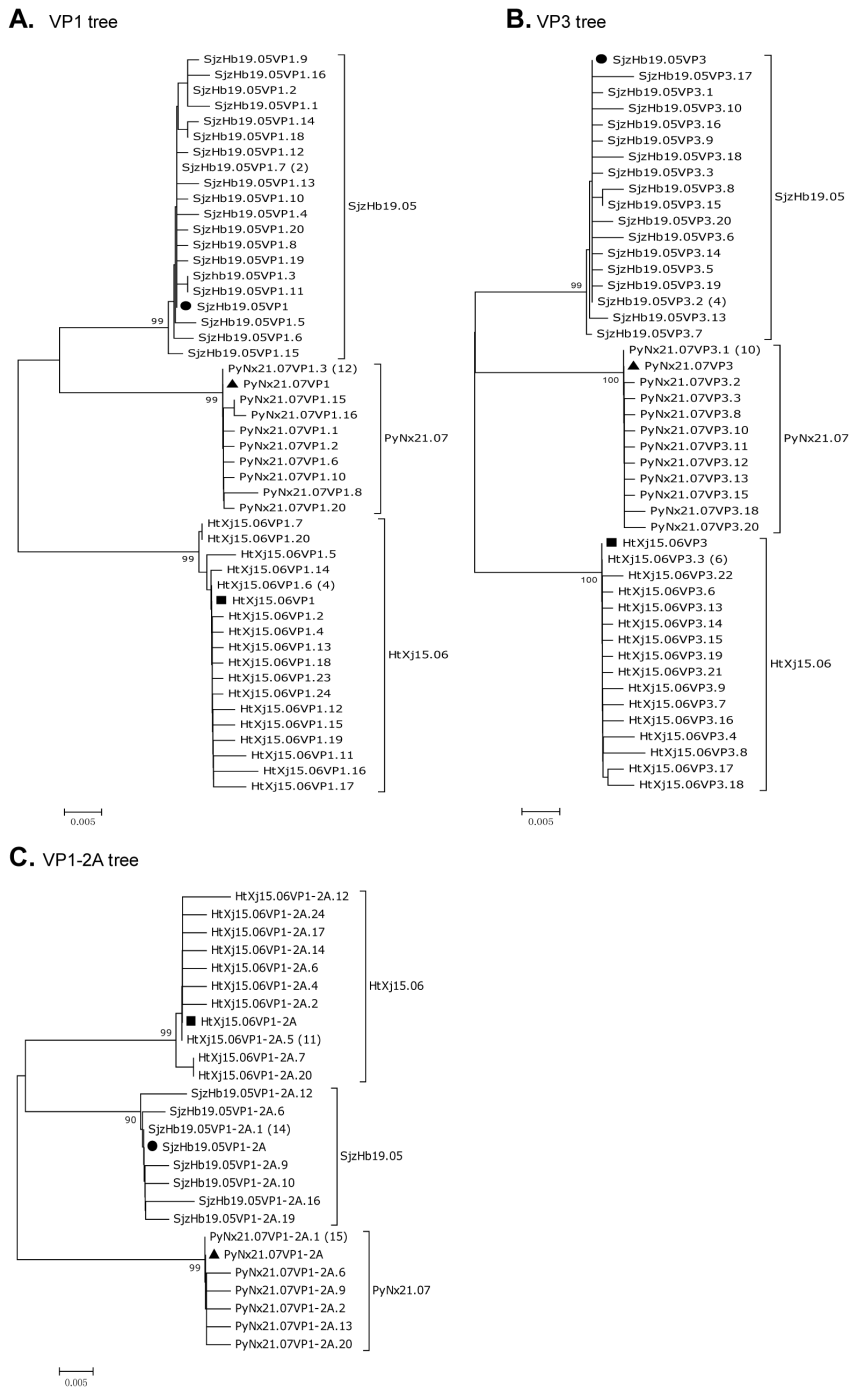
Quasispecies analysis of HAV isolates from three clinical samples. Phylogenetic tree of the nucleotide mutant spectra of different clones. The complete VP1 (A), complete VP3 (B) and VP1-2A junction region (C) were analyzed. Consensus sequences for SjzHb19.05 (●), HtXj15.06 (■), or PyNx21.07 (▲). Numbers in parentheses indicate clones identical to the consensus sequences. The neighbor-joining method under the Kimura-two parameter distance model was employed. Numbers at the branches show bootstrap percentages obtained after 1000 replications of bootstrap sampling. Bars indicate distances. The heterogeneity of the clones can be seen from the trees.

For each isolate, the clones exhibited an intra-isolate nucleotide sequences identity of more than 98.7%, and amino acid sequences identity of more than 96.1% for the VP1-2A junction region, complete VP1 and VP3 genes respectively. As shown in Figure 2, most of the clones differed at only one nucleotide (or amino acid) site, but at different positions. Some clones contained several deletions (e.g. in SjzHb19.05 or HtXj15.06 clones), or insertions (e.g. in PyNx21.07 clones), leading to a heterogeneous variety of clones per isolate. All instances of mutation from the consensus sequence are presented in only a minority of the samples analyzed.

The Shannon entropies (S_N_) of the sequenced quasispecies were calculated to assess the quasispecies complexity, where 0 indicates no diversity and 1 indicates maximum diversity. Nucleotide sequence (S_N_) values ranged from 0.38-0.98, and amino acid sequence (S_NA_) values ranged from 0.26-0.77, indicating heterogeneity of the mutant spectra.

The nucleotide mutation frequency ranged from 7.22x10^-4^ to 2.33x10^-3^ substitutions per nucleotide, and the amino acid mutation frequency ranged from 1.55x10^-3^ to 5.04x10^-3^ per amino acid in the complete VP3, VP1 or VP1-2A junction regions of the isolates studied. These results are comparable with the published mutation frequencies [15,17], and the quasispecies diversity confirms previous reports of the heterogeneity of circulating HAV strains [28,29,30,31]. Table 4. Characterization of the mutant spectrum of HAV isolates from clinical samples in this study.

**Table 4:** Characterization of the mutant spectrum of HAV isolates from clinical samples in this study.

Genomic region/Isolates	Mutations^a^/Nucleotides sequenced	Nucleotide mutation frequency^b^	Amino acid mutation frequency^b^		
				S_N_^C^	S_NA_^d^
VP3/HtXj15.06	26/14760	1.76x10^-3^	3.25x10^-3^	0.82	0.72
VP3/PyNx21.07	14/14760	9.49x10^-4^	1.63x10^-3^	0.67	0.50
VP3/SjzHb19.05	29/14760	1.96x10^-3^	2.44x10^-3^	0.91	0.56
VP1/HtXj15.06	37/18000	2.06x10^-3^	3.17x10^-3^	0.95	0.77
VP1/PyNx21.07	13/18000	7.22x10^-4^	1.83x10^-3^	0.56	0.50
VP1/SjzHb19.05	36/18000	2.00x10^-3^	2.67x10^-3^	0.98	0.77
VP1-2A/HtXj15.06	18/7740	2.33x10^-3^	5.04x10^-3^	0.75	0.59
VP1-2A/PyNx21.07	6/7740	7.75x10^-4^	1.55x10^-3^	0.38	0.26
VP1-2A/SjzHb19.05	16/7740	2.07x10^-3^	3.88x10^-3^	0.59	0.45

^a^ Mutant residues are those that vary relative to the corresponding consensus sequences

^b^ The nucleotide mutation frequency is the total number of mutations divided by the total number of nucleotide sequenced. The amino acids mutation frequency is the total number of nonsynonymous mutations divided by the number of amino acids encoded in the sequence analyzes.

^C^ The normalized Shannon entropy is calculated as S_N_= - [∑i (pi lnpi)] /lnN, in which pi is the proportion of each sequence of the mutant spectrum and N is the total number of sequences compared.

^d^ The normalized Shannon amino acid entropy was calculated as S_NA_= - [∑i (qi lnqi)] /lnN, where q_i_ is the frequency of each amino acids sequence of the mutant spectrum and N is the total number of sequences compared.

## Discussion

In this paper, part of the hepatitis A outbreak cases with identical sequences in the VP1-2A junction region reported from 2003 to 2010 in China were selected for further analysis in the complete VP3-VP1-2A regions. Results indicated the circulation of both sub-genotype IA and IB in the country, with genotype IA most prevalent. Based on the definition of Robertson [13], the same genotyping results can be obtained whether using the VP1-2A junction region or the complete VP1 or VP3-VP1-2A regions in this study, although some differences did exist. HAV isolates from the same area tend to clustered to one of several closely related lineages, suggesting either that these particular sub-genotypes possess a fitness advantage in the region, or an endemic transmission pattern of closely related strains co-circulating in these regions [32,33,34,35].

For some isolates collected from the same year and area, e.g. SjzHb17.05 and SjzHb19.05, the patients studied appeared to be infected with HAV strain identical in the VP1 and VP3 regions. This suggests a common source of infection [34,36,37]. In other cases such as the closely related PyNx21.07 and PyNx26.07 isolates, samples share identical VP1-2A sequences, but sequencing of the entire VP1 and VP3 genes identified minor nucleotide polymorphisms. The variable homogeneity of the isolates may reflect diversity in infection source. Some sources of infection may be contaminated with several different well-adapted HAV strains co-circulating in the same geographic area, previous reports have made similar observations [4,19,38,39].

Some isolates collected from different areas during different years share identical VP1-2A junction sequences, but differ by several nucleotides and amino acids in the complete VP1 and VP3 genes, e.g. HtXj2.06 and PyNx11.07. This genetic diversity was greater than that between those isolates collected at the same site during the same year in this study. It is likely that the virus was derived from travellers coming from HAV endemic areas, however, it may also be the case of the endemic of the virus, that several different HAV strains with similar VP1-2A sequences co-circulating in different geographic areas [18,34]. Alternatively the genetic diversity presented at each site might be a result of locally generated mutant spectrum in the course of each HAV outbreak, probably due to the presence of different selective pressures, such as virus variants to respond to host defense mechanisms or to favor replication in the face of physiological alterations [17]. Detailed epidemiological investigations and the full genomic analysis may elucidate the mechanisms underlying this phenomenon [40,41,42].

HAV is a virus known to demonstrate low antigenic variability, as reflected by the existence of a single serotype [7,43]. In this study, several amino acid differences were found in the analyzed VP3-VP1 coding regions from the consensus sequences of the Chinese HAV isolates and the published reference sequences, none were found at the published neutralizing antigenic sites (Table 3) [7,9,10], but two replaced amino acid positions (LpXj1.06, VP1-115 and HtXj25.06, VP1-164) were found around the viral immunodominant site, while mutations at position VP1-166 have been detected in MSM patients who had been partly vaccinated [20]. Thus further analysis of the possibility of antigenic escaping mutants should be carried out in the future.

Viral natural selection can be positive (or adaptive, i.e. selection for mutants that have advantageous trait) or negative (also known as purifying, i.e. the removal of mutants that have disadvantageous trait); if there is absence of any natural selection, the viral gene is evolving purely by genetic drift [27]. Nucleotide substitution in protein-coding regions can be classified as non-synonymous (dN) and synonymous (dS). Non-synonymous (dN) substitutions in a gene result in alterations in the translated amino acid sequence, thus are more likely than synonymous (dS) substitutions to alter protein function. We found both synonymous and non-synonymous mutation in the HAV isolates VP3-VP1 capsid sequences, and the overall calculation of dN/dS<1, indicated that HAV is undergoing negative selection. The greater conservation of amino acids within HAV VP3-VP1 is probably due to the rare codon usage and the structure constrains in the viral capsid which limit the amino acids variability [15,16,17].

Analysis of the VP3 and VP1 coding regions revealed that closely genetically related variants of HAV, termed quasispecies, are circulating in China. The population is more complex than we anticipated. For example although many clones differ by only one nucleotide or amino acid, these differences occur at a variety of sites within the genome, producing a heterogenous group of clones. At each mutation site analyzed the majority of clones match the consensus sequence, and mutated nucleotides were all in the minority. The nucleotide mutation frequencies in this study are about 7.22x10^-4^ to 2.33x10^-3^ for VP3, VP1 or VP1-2A gene regions, similar to previous reports [15,17,28,30,44]. This mutation rate is significantly lower than that for other members of the Picornaviridae family (for example, 2.7x10^-2^ for FMDV, 3.36x10^-2^ for poliovirus type 1, 2.2x10^-2^ for enterovirus 70) [30]. Many authors have reported the existence of quasispecies in HAV infected individuals, and it may be possible that the mutated genomes which constitute a minority species in each infected individual become dominant after transmission to a new host individual [17,28,29]. This mechanism could in part explain the observed closely related isolates obtained in this study.

In conclusion, this study analyzed the VP3-VP1-2A genes of HAV sequences from selected hepatitis A outbreak cases collected from different areas during different years in China. HAV is under negative selection. Diverse HAV strains and quasispecies inside the viral populations are presented in China, with unique amino acid substitutions detected close to the immunodominant site. The possibility of antigenic escape mutants cannot be ruled out and needs to be further analyzed. In the future, more HAV isolates and full length genome analysis, additional molecular approaches and detailed epidemiological investigation of HAV from the country would be taken into account for a better understanding of the genetic diversity of HAV in China.
